# Neuropeptide G Protein-Coupled Receptors as Oncotargets

**DOI:** 10.3389/fendo.2018.00345

**Published:** 2018-06-29

**Authors:** Terry W. Moody, Irene Ramos-Alvarez, Robert T. Jensen

**Affiliations:** ^1^Department of Health and Human Services, National Cancer Institute, Center for Cancer Research, National Institute of Diabetes, Digestive, and Kidney Disease (NIDDK), Bethesda, MD, United States; ^2^Digestive Diseases Branch, National Institute of Diabetes, Digestive, and Kidney Disease (NIDDK), Bethesda, MD, United States

**Keywords:** cancer GPCR, cancer RTK, bombesin, neurotensin, vasoactive intestinal peptide, pituitary adenylate cyclase activating polypeptide (PACAP), somatostatin, cancer signal transduction

## Abstract

Neuropeptide G protein-coupled receptors (GPCRs) are overexpressed on numerous cancer cells. In a number of tumors, such as small cell lung cancer (SCLC), bombesin (BB) like peptides and neurotensin (NTS) function as autocrine growth factors whereby they are secreted from tumor cells, bind to cell surface receptors and stimulate growth. BB-drug conjugates and BB receptor antagonists inhibit the growth of a number of cancers. Vasoactive intestinal peptide (VIP) increases the secretion rate of BB-like peptide and NTS from SCLC leading to increased proliferation. In contrast, somatostatin (SST) inhibits the secretion of autocrine growth factors from neuroendocrine tumors (NETs) and decreases proliferation. SST analogs such as radiolabeled octreotide can be used to localize tumors, is therapeutic for certain cancer patients and has been approved for four different indications in the diagnosis/treatment of NETs. The review will focus on how BB, NTS, VIP, and SST receptors can facilitate the early detection and treatment of cancer.

## Introduction

G protein-coupled receptors (GPCRs) have 7 transmembrane (T M) domains and they interact with G proteins comprised of α, β, and γ subunits (1). The activated GPCRs undergoes a conformation change dissociating the G-protein into a GTP-bound α subunit and β, γ dimer. GPCRs for bombesin (BB) and neurotensin (NTS) interact with Gq/11, whereas receptors for vasoactive intestinal peptide (VIP) interact with Gs and somatostatin (SST) receptors interact with Gi/0 (2). BB and NT receptors cause phosphatidylinositol (PI) turnover resulting in the elevation of cytosolic Ca^2+^ and activation of protein kinase (PK) C. VIP receptors activate adenylyl cyclase resulting in elevated cAMP whereas SST receptors reduce the elevation of cAMP stimulation caused by VIP.

Neuropeptides modulate neural activity in the brain in a paracrine manner, however, they function as autocrine growth factors in cancer (3). BB, NTS, and VIP stimulate the growth of small cell lung cancer (SCLC) cells whereas SST inhibits growth (3). BB and the structurally related gastrin-releasing peptide (GRP) bind with high affinity to the GRP receptor or BB_2_R; NTS binds high affinity to NTSR1; VIP binds with high affinity to VPAC1/VPAC2 and SST as well as octreotide/lanreotide bind with high affinity to SSTR2/SSTR5 but reduced affinity to SSTR1, SSTR3, and SSTR4. The agonist-GPCR complex is internalized and the GPCR recycle to the membrane but the peptide is metabolized in lysosomes. Because cancers frequently over-express GPCRs, the cancer GPCRs can be used to deliver neuropeptide-drug conjugates into the cancer cell (4). In contrast, GPCR antagonists bind to the GPCR at the cell surface but are not internalized. PD176252 (GRPR antagonist) and SR48692 (NTSR1 antagonist) inhibit cancer growth (5–7). This review will focus on how neuropeptide GPCRs are oncotargets for the early detection and treatment of cancer.

## GRPR, neuromedin B receptor and BBR subtype 3

SCLC has high levels of the BB-like peptide GRP (8, 9). GRP is derived from a 148 amino acid prepropeptide (10). After removal of the N-terminal 23 amino acid signal sequence, pro-GRP^1−125^ is metabolized by a prohormone convertase to GRP, which contains 27 amino acids and has an amidated C-terminal. GRP (proGRP^1−27^) is readily metabolized in the blood but SCLC patients have elevated pro-GRP (11). Antibodies to proGRP^31−98^ have been used to detect high concentrations of proGRP (>100 pg/ml) in the serum of patients with SCLC. Because proGRP is elevated in the serum of 71% of the SCLC patients it may be a biomarker for SCLC (12). BB or GRP, but not proGRP bind with high affinity to the GRPR. The C-terminal octapeptide of BB or GRP can be neutralized by mAb 2A11. mAb2A11 inhibits the growth of SCLC *in vitro* and in mouse models *in vivo* (13). In a clinical trial, 2A11 was well tolerated and one patient had SCLC remission whereas four patients had stable disease out of 13 patients treated (14). The results indicate that the GRP precursor may be a biomarker for SCLC.

Table 1 shows that the GRPR, which is localized to chromosome xp22, contains 384 amino acids and is a member of the class A/Rhodopsin-like GPCR (15, 16). The neuromedin B (NMB) R or BB_1_R, which is localized to chromosome 6q24, contains 390 amino acids whereas BB receptor subtype-3 (BRS-3), which is localized to chromosome xq26, contains 399 amino acids. The NMBR and BRS-3 have about 50% sequence homology with the GRPR (17, 18). The GRPR binds GRP and NMB with high and low affinity, respectively. The NMBR binds GRP and NMB with low and high affinity, respectively. The orphan receptor BRS-3 binds both GRP and NMB with low affinity but MK5046 binds with high affinity (7). The universal agonist BA1, (D-Tyr^6^, β-Ala^11^, Phe ^13^, Nle^14^) BB^6−14^, binds with high affinity to the GRPR, NMBR, and BRS-3. Numerous amino acids in TM domains 6 and 7 as well in extracellular loops (EL) 1, 2, and 3 of the GRPR are essential for high affinity binding of GRP (4). While the GPCRs of each family have a similar sequence, the pharmacological profile is different.

**Table 1:** Peptide GPCRs (human).

**Receptor**	**GRPR**	**NMBR**	**BRS-3**	**NTSR1**	**NTSR2**	**VPAC1**	**VPAC2**	**PAC1**
Chromosome	xp22	6q24	xq26	20q13	2p25	3p22	7q36	7p14
Amino acids	384	390	399	418	410	457	438	468
G-protein	Gq	Gq	Gq	Gq	Gq	Gs	Gs	Gs, Gq
Agonist	BB, GRP	NMB	MK5046	NTS	NTS	VIP	VIP	Maxidillin
	BA1	BA1	BA1	JMV449	Levocabastine	PACAP	PACAP	PACAP
						(Lys^15^, Arg^16^, Leu^17^)VIP^1−7^GRF^8−27^	R025-1553	
Antagonist	RC3095	PD168368	Bantag1	SR142948A	SR142948A	VIPhyb	VIPhyb	PACAP(6–38)
	(Psi^13, 14^, Leu^14^)BB			SR48692				
	PD176252							

BB-drug conjugates were synthesized which are cytotoxic for lung cancer cells. The topoisomerase-1 inhibitor camptothecin (CPT) was coupled with a linker to the N-terminal of BA1. Surprisingly, the resulting CPT-L2-BA1 bound with higher affinity to the GRPR, NMBR, and BRS-3 than did BA1 (19). CPT-L2-BA1 was an agonist which increased PI turnover and was internalized. The CPT-L2-BA1 was metabolized in the lysosome leading to the release of CPT (20). Also, BB agonists have been coupled to paclitaxel (21), doxorubicin (22), marine toxins (23), magainin II (24), and siRNA to the GRPR (25) resulting in decreased cancer cellular proliferation. Doxorubicin was coupled to a GRPR antagonist and the resulting AN-215 was cytotoxic for gastric, colon, lung, ovarian, endometrial, breast, and pancreatic cancer (26). RC-3095, a GRPR antagonist, was tested in 25 patients with solid tumors. RC-3095 had minimal toxicity but a partial response was only seen in 1 patient (27). Unfortunately, these BB-drug conjugates will not only kill cancer cells, but normal cells with excessive BBR.

BBR antagonists were developed which inhibit the growth of cancer cells. Peptide antagonists such as RC-3095 or (Psi^13, 14^, Leu^14^)BB blocked the GRPR, and they inhibited the growth of cancer cells (27, 28). Small molecule antagonists such as PD168368 were synthesized which inhibit the growth of cancer cells which have NMBR (6). Also, bantag-1 is a peptide antagonist for BRS-3 (29). The BB receptor antagonists inhibited the growth of lung cancer cells *in vitro* and *in vivo* using nude mice bearing lung cancer xenografts. GRPR, NMBR, and BRS-3 mRNA was detected in 11/13 lung cancer cell lines (7). All lung cancer cell lines tested had at least 1 type of BBR and many cell lines had all 3 receptors.

In contrast, a high density of GRPR but not NMBR or BRS-3 were detected in most prostate and breast cancer cells (30). GRPR agonists were labeled with ^111^In, ^64^Cu, ^99m^Tc, ^68^Ga, ^18^F for imaging studies. Using a ^99m^Tc-BB^2−14^ analog 14 prostatic lesions were visualized in patients (31). Using a ^99m^Tc-RGD-BB analog, tumors were visualized in 6/6 breast cancer patients (32). Using a ^64^Cu-BB^6−14^ analog, tumors were visualized in 3 of 4 prostate cancer patients (33). It remains to be determined if the imaging of GRPR will be useful in the early detection of breast and/or prostate cancer.

**Figure 1:**
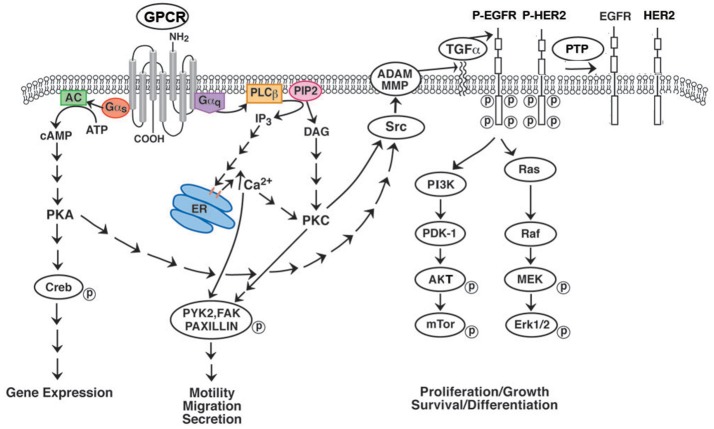
Effect of GPCR's on RTK transactivation. GPCRs for BB and NTS couple to Gq and causes metabolism of PIP_2_ to DAG (activates PKC) and IP_3_ (elevates cytosolic Ca^2+^). Addition of NTS or BB to NSCLC cells increases phosphorylation of PYK2, FAK or paxillin leading to increased cellular migration. GPCR for VIP interact with Gs activating adenylyl cyclase and increasing cAMP. The cAMP activates PKA leading to CREB phosphorylation and altered gene expression. GPCR for PACAP interact with both Gq and Gs. GPCR activate Src and MMP resulting in the production of EGFR ligands such as TGFα. When TGFα binds to the EGFR, tyrosine kinase activity is increased leading to phosphorylated EGFR homodimers or EGFR-HER2 heterodimers. The RTK activates the Ras-Raf-MEK-ERK pathway leading to increased cellular proliferation. The RTK activates the PI3K-PKD-AKT-mTOR pathway leading to increased cellular survival. The phosphorylated EGFR/HER2 is dephosphorylated by protein tyrosine phosphatase (PTP).

Many of the growth effects of BB-like peptides on non-SCLC (NSCLC) cells may result from transactivation of receptor tyrosine kinases (RTK) such as the epidermal growth factor receptor (EGFR). Activation of the NMBR in NSCLC cells causes PI turnover leading to increased phosphorylation of the EGFR (Figure 1). Addition of NMB to NSCLC cells increases the tyrosine phosphorylation of the EGFR after 1 min leading to the tyrosine phosphorylation of ERK after 2 min (34). The transactivation of the EGFR that is regulated by the NMBR is inhibited by the tyrosine kinase inhibitor (TKI) gefitinib or the NMBR antagonist PD168368. The transactivation process in NSCLC cells is mediated by the EGFR ligand transforming growth factor (TGF)α (Figure 1). The inactive precursor pro-TGFα is metabolized by matrix metalloprotease (MMP) enzymes in the membrane to biologically active TGFα which is secreted and binds to the EGFR. The transactivation of the EGFR caused by addition of NMB to NSCLC cells is inhibited by GM6001 (MMP inhibitor) or anti-TGFα Ab. The transactivation process requires reactive oxygen species (ROS). Addition of N-acetyl cysteine (antioxidant) or tiron (superoxide scavenger) impaired the ability of NMB to increase EGFR tyrosine phosphorylation. The ROS may oxidize Cys^773^ of the EGFR increasing its tyrosine kinase activity and/ or oxidize protein tyrosine phosphatases (PTP) impairing their ability to metabolize phosphotyrosine (35, 36). The results indicate that GPCRs regulate the transactivation of receptor tyrosin kinases (RTKs) in NSCLC cells.

The EGFR contains 1,186 amino acids with a 621 and extracellular domains I and III bind EGF or TGFα with high affinity (37). The EGFR has a 23 amino acid TM domain and a 542 intracellular domain with tyrosine kinase activity. Lys^721^ is essential for binding ATP and the phosphorylation of protein substrates. Upon binding of ligand, the EGFR can undergo homodimerization resulting in the phosphorylation of Tyr^1068^, Tyr^1086^, Tyr^1148^, and Tyr^1174^. Alternatively, the EGFR can form heterodimers with HER2. The MAPK and PI3K/Akt pathways are downstream of the EGFR and are important for EGFR mediated proliferation and cancer cellular survival, respectively. Currently, we are investigating if GPCRs transactivate additional RTK such as HER2, HER3 or HER4 in cancer cells.

NMB increases the proliferation of NSCLC cells. In contrast, PD168368 and gefitinb inhibit the growth of NSCLC cells (34). Surprisingly, combinations of the NMBR antagonist with the EGFR TKI reduced the proliferation of NSCLC cells in a synergistic manner. The results indicate that GPCR antagonists potentiate the action of TKI in NSCLC. Traditionally NSCLC which kills 130,000 U.S. citizens annually is treated with combination chemotherapy, however, the 5 year survival rate is only 15%. The EGFR is mutated is approximately 13% of the NSCLC patients and those with the L858R mutation have increased tyrosine kinase activity and sensitivity to TKI such as gefitinib or erlotinib (38, 39). Traditionally NSCLC patients are treated with combination chemotherapy, however, the 5 year survival rate is only 15% (40).

## Neurotensin receptors

NTS is present in numerous SCLC cell lines (9, 41). NTS is derived from a 170 amino acid precursor and metabolized to a biologically active peptide which contains 13 amino acids (42). NTS and its C-terminal fragment NTS^8−13^ bind with high affinity to the NTSR1, which is localized to chromosome 20q13, contains 418 amino acids, and is a class A/Rhodopsin-like GPCR. Table 1 shows that the NTSR2 which is on chromosome 2p25 contains 410 amino acids and binds NTS and levocabastine with high affinity. The NTS^−^-NTSR1 complex has been crystallized and NTS^8−13^ sits on top of the NTS binding pocket and interacts with TM domains 6 as well as 7 and EL 2 as well as 3 (43). Both NT and BB receptors have short N-terminals which have little effect on ligand binding (44). The nonpeptide NTSR1 antagonist SR48692 binds deep into the NTSR1 binding pocket and blocks the effects of NTS agonists. Also, SR142948A blocks both the NTSR1 and NTSR2. The NTSR3 is not a GPCR but is sortilin.

NTS binds with high affinity to SCLC cells (45). Addition of NTS to cancer cells causes PI turnover leading to increases PKC activity and elevation of cytosolic Ca^2+^ (46–48). In contrast, the NTSR2 agonist levocabastin has little effect on lung cancer cells. The effects of NTS on second messenger production and proliferation was antagonized by SR48692 (5). NTS addition to cancer cells causes phosphorylation of various proteins such as focal adhesion kinase (FAK) or ERK (49, 50). The phosphorylated ERK increases the expression of c-fos and c-jun leading to cellular proliferation (51). NTS stimulates proliferation whereas SR48692 inhibits the proliferation of lung cancer cells (5). NT addition to NSCLC cells increased EGFR tyrosine phosphorylation 5-fold (52). NT (5 nM) half-maximally increased EGFR transactivation after 2 min. NTS or NTS^8−13^ but not NT^1−8^ or levocabastine increase EGFR tyrosine phosphorylation. The NTSR1 regulation of EGFR transactivation is inhibited by SR48692, gefitinib, PP1, GM6001, TGFα antibodies and antioxidants. SR48692 and gefitinib inhibit the proliferation of NSCLC cells in a synergistic manner. Previously, JMV449, a NT^8−13^ analog, was found to increase expression of the EGFR, HER2, and HER3 after 24 h (53). JMV449 addition to cells increase MMP activity resulting in HB-EGF and neuregulin 1 release, which activates the EGFR and HER3, respectively.

NTSR1 regulates the EGFR transactivation in numerous cancers including colon, foregut neuroendocrine, lung, and prostate cancer (47, 52, 54, 55). Lung cancer and gastric cancer patients whose tumors had high densities of NTSR1 had decreased survival (53, 56). Addition of NTS to NSCLC cells caused tyrosine phosphorylation of the EGFR in a PLC-dependent manner (52). Phosphorylated β-catenin dissociates from E-cadherin and increases the expression of NTSR1. Wnt/β-catenin signaling increases the expression of E-cadherin leading to epithelial to mesenchymal transitions and cancer metastasis (57). Recently, 3BP-227, a SR142948A analog, was radiolabeled and used to image tumors containing NTSR1. In nude mice containing HT29 colon cancer tumors ^177^Lu-3BP-227 localized to the tumors with high tumor-to-kidney or tumor-to-liver ratios using whole-body SPECT/CT techniques (58). In 5 out of 6 patients with ductal pancreatic adenocarcinoma tumor uptake of ^177^Lu-3BP-227 was observed (59). It remains to be determined if ^177^Lu-3BP-227 will improve survival of patients whose tumors are enriched in NTSR1.

## VIPRs and pituitary adenylate cyclase activating polypeptide receptor

The biological activities of the VIP and PACAP family of peptides are mediated by 3 GPCR (VPAC1, VPAC2, and PAC1), which are members of the classB/secretin-like receptors (60). Table 1 shows that VPAC1, which is localized to chromosome 3p22, contains 457 amino acids with a 112 amino acid N-terminal. VPAC2, which is localized to chromosome 7q36, contains 438 amino acids with a 103 amino acid N-terminal. PAC1, which is localized to chromosome 7p14, contains 468 amino acids with a 125 amino acid N-terminal. PAC1 has about 50% sequence homology with VPAC1 or VPAC2 (60). The large N-terminal extracellular domain of PAC1 has antiparallel β-sheets and binds to the C-terminal of PACAP (61, 62). The PAC1 receptor has 3 closed transitional states (G1-G3) and one open state named G4 (63). The N-terminal of PACAP, which activates PAC1 binds to EL and TM domains (64). VPAC1, VPAC2, and PAC1 interact with Gs resulting in elevated cAMP, however, PAC1 interacts with Gq as well resulting in PI turnover (65). VIP, which contains 28 amino acids, is derived from a 170 amino acid precursor protein. PACAP-27 as well as PACAP-38 is derived from a 176 amino acid precursor protein and 67% of the amino acids in PACAP-27 and VIP are identical (60). VPAC1 and VPAC2 binds VIP and PACAP-27 or PACAP-38 with high affinity, whereas PAC1 binds PACAP-27 or PACAP-38 with high affinity but VIP with low affinity. Maxidillin, a 61 amino acid peptide isolated from sand fly, binds with high affinity to PAC1 but not VPAC1 or VPAC2 (66). Recently, a number of PACAP-38 analogs were synthesized which prefer PAC1 relative to VPAC1 or VPAC2 by over an order of magnitude (67). VIPhybrid is a peptide antagonist which binds with moderate affinity to VPAC1 or VPAC2, whereas, PACAP(6–38) is a peptide antagonist for PAC1 (68). Selective non-peptide antagonists for VPAC1, VPAC2 or PAC1 remain unknown.

VPAC1 is present in numerous cancers including breast, colon, liver, lung, neuroblastoma, pancreatic, and prostate cancers in high densities (69). VPAC2 is present in moderate densities in gastric pancreatic adenocarcinomas, gastric leiomyomas, thyroid cancer, and sarcomas (70). PAC1 is present in brain, breast, colon lung, neuroendocrine, pancreatic, pituitary, and prostate cancer as well as neuroblastoma/pheochromocytoma (71). In SH1SY5Y neuroblastoma cells, numerous PAC1 splice variants (SV) were detected in the N-terminal and intracellular loop (IL) 3 (72). PAC1 has 18 exons and deletion of exons 5,6 or 4-6 reduce the N-terminal by 7, 21 (short) or 57 amino acids (very short) (73). The short PAC1 but not the very short PAC1 bind PACAP-38 with high affinity and elevate cAMP (74). Alternative splice variants (SV) of IL3 result in the addition of an additional 28 amino acid segment (hip) to PAC1null (75). Addition of a different set of 28 amino acids to IL3 of the PAC1 results in the hop SV. Finally, both SVs can be added resulting in PAC1hiphop. The order of potency to increase PI turnover was PAC1hop > PAC1null = PAC1hiphop > PAC1hip (76). Thus binding of PACAP and second messenger production can be altered by PAC1 deletions and SVs.

VPAC1 can be utilized to image cancer tumors. ^18^F(Arg^15, 21^)VIP localized to T47D breast cancer cells in nude mice (77) and ^64^Cu-TP3982 localized to mammary tumors in MMTVneu transgenic mice (78). ^99m^Tc-TP3982 was used to image breast tumors in 5 patients (79). The VPAC1-agonist complex internalizes in cancer cells and the ligand is metabolized in lysosomes. VPAC1 has been used to deliver VIP analogs containing cytotoxic CPT, paclitaxel, ellipticin or geldanomycin to cancer cells (80–83). The actions of VIP are antagonized by peptides such as VIP^10−28^ or VIPhybrid (84). Addition of VIP to cancer cells results in elevated cAMP which activates PKA. Activation of PKA results in CREB phosphorylation which increases nuclear oncogene expression of c-Myc leading to increased proliferation (Figure 1). VIP increases the proliferation of lung cancer cells whereas VIPhybrid inhibits proliferation (84). Addition of PACAP-27 or PACAP-38 to lung cancer cells containing VPAC1, VPAC2 or PAC1 increases cAMP, however, it causes PI turnover in cells containing PAC1. When PI is metabolized, ERK becomes phosphorylated. Phosphorylated ERK increased the expression of the nuclear oncogenes c-fos and c-jun leading to increased cancer cellular proliferation. PACAP(6–38) inhibits the proliferation of lung cancer cells *in vitro* and *in vivo* (85).

VIP may be a promoter of carcinogenesis. VPAC1 density is higher in mammary cancer than adjacent normal tissue using rat and mouse models (86). Specific binding of ^125^I-VIP to mouse mammary tumors was inhibited with high affinity by (Lys^15^, Arg^16^, Leu^17^) VIP^1−7^GRF^8−27^ (VPAC1 peptide agonist) but not Ro25-1553 (VPAC2 peptide agonist). Retinoic acid, a chemopreventive agent, down-regulates VPAC1 expression in breast and lung cancer cells (87, 88). Finally VIPhybrid inhibits mammary carcinogenesis in C3(1)SV40T antigen mice (89).

Addition of PACAP-27 or PACAP-38 but not VIP causes transactivation of the EGFR in NSCLC cells (90). The PAC1 regulation of EGFR tyrosine phosphorylation is inhibited by PACAP(6–38), gefitinib, PP2, GM6001, and ROS inhibitors. Diphenyleneiodonium (DPI), a NADPH oxidase (NOX) inhibitor impaired the ability of PACAP to increase EGFR tyrosine phosphorylation. NOX-4, which produces ROS, is present in NSCLC cells (91). PACAP-27 addition to NSCLC cells increased ROS which was inhibited by DPI. VIP addition to breast cancer cells increased EGFR and HER2 phosphorylation (92). The EGFR which dimerizes, may form homodimers with itself or heterodimers with HER2. VPAC1 regulation of EGFR transactivation was blocked by JV-1-53 (VPAC1 antagonist), PP2 or H89 (PKA inhibitor). In contrast, the PAC1 regulation of EGFR transactivation in NSCLC cells was inhibited by U73122 (phospholipase C inhibitor) but not H89. The results indicate that the EGFR can be transactivated by GPCR which interact with Gq or Gs.

## SST receptors

SST occurs endogenously in two principal forms (SST-14, SST-28) and their action is mediated by 5 related subtypes of GPCRs (SSTR1-5) (93, 94). SST receptors are not only widely expressed on normal tissues, but also are frequently overexpressed by many neoplasms, particularly NETs [i.e., carcinoids/pancreatic neuroendocrine tumors (panNETs)] (93–95). SST has a wide range of physiological actions and they are primarily inhibitory (93, 94).

SST and its receptors represent the prototype for a clinically successful peptide/peptide receptor oncotarget. It is the only peptide-GPCR system which has multiple approved indications (four different indications) for the treatment of a class of human neoplasms, NETs. Furthermore, its results in NETs have potential applicability for its clinical utility in a number of other neoplasms.

**Figure 2:**
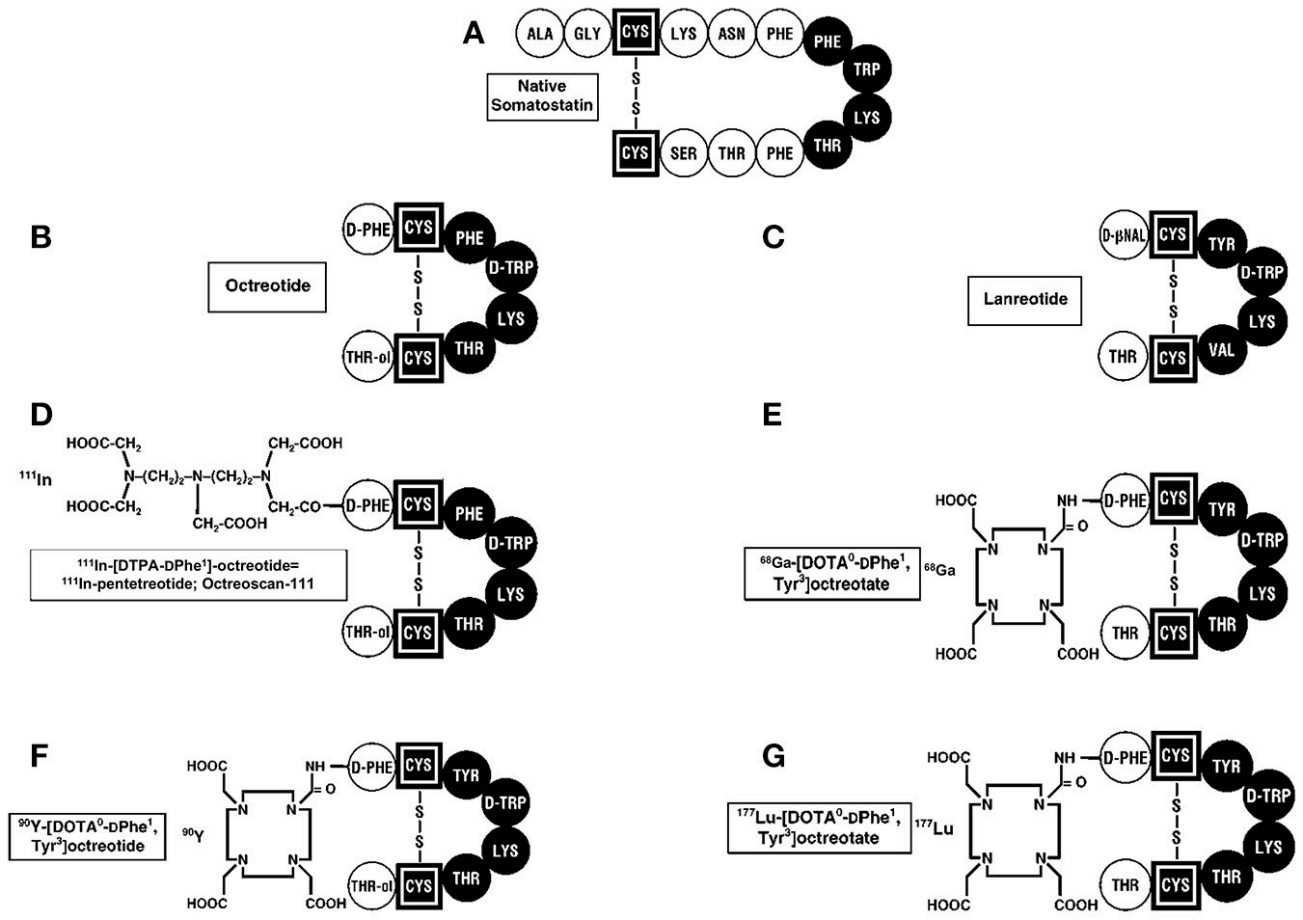
Structures of SST and synthetic analogs used clinically. **(A)** The 14 amino acid SST is shown and essential amino acids are in black. The SST synthetic analogs, octreotide **(B)**, and lanreotide **(C)**, which have 8 amino acids, are approved to treat patients with neuroendocrine tumors (NETs) producing hormone-excess states (VIPoma, carcinoid syndrome) and for their anti-proliferative activity in patients with advanced, aggressive NETs. ^111^InDTPA pentetreotide **(D)** as well as ^68^GaDOTATATE **(E)** are used for SSTR imaging in patients with NETs. ^90^Y-DOTA **(F)** and ^177^Lu-DOTA **(G)** -labeled SST analogs are used for their antitumor activity during PRRT by targeting the cytotoxic radiolabel to the tumor.

The initial approved indication for SST analogs was its use in hormone-excess states. Depot long-acting formulations of synthetic SST analogs (octreotide-LAR, lanreotide autogel) (Figure 2) are the drugs of choice to control various functional NET syndromes due to the ectopic release of a biologically active peptides by the NETs (93, 96–99). This includes the control of such widely different functional NET syndromes as the carcinoid syndrome (diarrhea, flushing) due to metastatic carcinoid tumor; severe diarrhea due to VIPomas; rash due to glucagonomas; acromegaly due to excessive growth hormone release primarily by pituitary adenomas, and a number of others (93, 96–100). Almost all (>90%) of the well-differentiated forms of these NETs (>95%) overexpress somatostatin receptor subtype 2 which has high affinity for octreotide and therefore it is generally effective in controlling the hormone-excess state and in many, their growth. Some NETs such as pituitary adenomas do not overexpress SSTR2 with the result that octreotide/lanreotide are only effective in 20–70% of patients (101). One solution to this has been the development of next generation somatostatin analogs such as pasireotide, which has high affinity for multiple subtypes (SSTR5 > SSTR2 > SSTR3 > SSTR1), which has been shown to be effective in these patients and is approved for use in the treatment of a acromegaly.

The second approved indication for SST analogs is for its anti-proliferative activity on NETs. In numerous pre-clinical studies and animal studies, it was shown that SST analogs have anti-proliferative effects on NETs, as well as number of other human tumors (102–107) Two double-blind Phase 3 studies (108, 109) in patients with advanced NETs treated with lanreotide/octreotide increased the patient's progressive free survival (PFS), which lead to FDA approval. Recent meta-analyses (106, 110) of SST analogs anti-proliferative effects in all publications (106) or the above two studies (110), in patients with advanced NETs, demonstrate good anti-proliferative activity, significant benefit from their use resulting in disease control (HR 0.51, *p* < 0.01), with the response rates vs. placebo being 58 vs. 32% and a good safety profile. In general, these studies demonstrate that SST analog treatment in patients with advanced NETs result in a tumoristatic effect primarily, rather than a decrease in the tumor size. SST analogs are now recommended in recent guidelines as well as expert reviews as one of the initial treatments for controlling tumor growth in patients with advanced NETs, especially those with well-differentiated NETs, and slower growth rates (99, 111–115). Numerous *in vitro* and animal studies report that SST receptors are expressed on a number of other non-endocrine tumors, and that SST analogs have anti-proliferative activity in these tumors (93, 94, 97, 103, 104, 107, 116, 117). No controlled trials have established the use of SST analogs for anti-proliferative effects in patients with non-endocrine tumors.

The third approved indication for SST analogs in patients with NETs is for imaging of the tumor. SST receptor imaging (SRI) was originally approved for the use of ^111^In-labeled pentetreotide with SPECT/CT scanning (Octreoscan), which is now replaced by the use of ^68^Ga-DOTATATE PET/CT scanning, which has greater resolution, sensitivity and high specificity (118–122). Almost all well differentiated NETs overexpress the somatostatin receptor subtypes (SSTR2 > SSTR5 > SSTR3) that bind this radiolabeled agonist with high affinity (118, 119, 123). A systematic review (122) demonstrated that the use of ^68^Ga-DOTATATE PET/CT scanning changed the management of the patient in a mean of 44% (range, 16–71%). SRI is now essential for the staging and management of NET patients and is the most sensitive method to allow whole body scanning rapidly to present a complete assessment of the extent of the tumor (112, 118, 121).

The fourth approved indication for SST analogs in patients with NETs is their use to target cytotoxic radiolabeled SST analogs to the tumor in patients with advanced NETs, as an anti-tumor therapy (called PRRT for peptide receptor radionuclide therapy) (124–127). Numerous animal studies as well as uncontrolled studies on patients with advanced NETs, demonstrated this approach resulted in tumor stabilization in progressive tumors as well tumor shrinkage in a significant number of patients with acceptable side-effects (124–127). Various SST analogs were coupled to linkers (DOTATATE, DOTATOC, DOTANOC) (Figure 2) and to different radiolabels including ^111^Indium,^90^ Yttrium, and ^177^Lutetium (127, 128). A recent double-blind controlled trial (129) demonstrated that ^177^Lutetium-DOTATATE treatment in patients with advanced midgut carcinoids, resulted in marked increase in progression-free survival and a preliminary result demonstrating increased overall survival, with acceptable safely profile. This has led to FDA approval for this treatment in patients with advanced NETs. Almost all of the early studies performed with SRI and PRRT used SST analogs that were agonists because of the belief the peptide should be internalized to provide the best imaging and radionuclide delivery to the tumor. Recent studies demonstrate that SST receptor antagonists recognize more binding sites on the tumor, provide superior tumor targeting to agonists (118, 130, 131) and also demonstrate greater membrane binding suggesting it will be superior for PRRT.

Unfortunately, the over-expression of SST receptors is limited to a subset of tumors and is not seen in many of the more frequent adenocarcinomas, such as breast, colon, lung or prostate and therefore the specific ligands developed for NETs will not be useful in these tumors. However, many of these other more common tumors over-express a number of other GPCRs including receptors for the BB, NTS, VIP/PACAP family (68, 132–134). Furthermore, in many cases selective ligands that are radiolabeled have been developed and studies in animal models and some cases in small numbers of humans with different diseases, support this approach (68, 132–134). Whether in the future they will become established for the imaging of these tumors or for the delivery of cytotoxic substances is unknown.

## Conclusions

Neuropeptide GPCR may play an important role in cancer proliferation, angiogenesis, and metastasis. Most of the research conducted on NTS, BB, and VIP has been at the preclinical level. NTSR1 and BBR are class A/Rhodopsin-like receptors which interact with Gq and cause PI turnover. Nonpeptide antagonists are available for the BBR and NTSR which inhibit the proliferation of cancer cells. NTS, BB, and VIP conjugates, which kill cancer cells, have been developed. BB, NTS or PACAP stimulate the proliferation of NSCLC cells in an EGFR-dependent manner. The transactivation of the EGFR is blocked by SR48692 (NTSR1 antagonist), PD176252 (GRPR antagonist) or PACAP(6–38) (PAC1 antagonist) as well as gefitinib (EGFR TKI). The GPCR antagonists potentiate the ability of TKI to reduce NSCLC growth *in vitro*. It remains to be determined if GPCR antagonists will potentiate the action of TKI *in vivo*. GPCR antagonists potentiate the effects of chemotherapeutic drugs (135, 136). VIPhybrid, a VPAC1 antagonist, potentiates the effects cytotoxicity of taxol in breast cancer *in vitro* and *in vivo*. Also, VIPhybrid potentiated the cytotoxicity of cisplatin, doxorubicin, gemcitabine, irinotecan or vinorelbine on colon cancer *in vitro*.

VIP and PACAP are class B/Secretin-like receptors which interact with Gs and stimulate adenylyl cyclase. PAC1 has numerous splice variants, which alter second messenger production. VIP has been coupled to radioisotopes, e.g., ^18^F, ^65^Cu, and ^99m^Tc to image tumors in animal models. High affinity non-peptide antagonists need to be developed for PAC1 VPAC1 and VPAC2. The use of peptide coated nanoparticles which contain chemotherapeutics is being investigated (137). The GPCR can be used to direct neuropeptide coated nanoparticle to the tumor. Recently, cholecystokinin antagonists were found to potentiate the effects of immune checkpoint inhibitors at impairing the growth of pancreatic tumors *in vivo* (138). Thus GPCR antagonists can potentiate the effects of various drugs in cancer treatment.

The use of SST analogs and their receptors are now an established part of clinical practice and provide the basis for 4 different FDA approved indications in patients with NETs. Long acting formulations of octreotide or lanreotide are the predominant therapeutic agents used to control excess secretion of peptides or growth hormone causing clinical syndromes in NET patients. Large clinical trials have recently led to the FDA approval of lanreotide/octreotide analogs to reduce NET growth in patients with advanced disease resulting in increased progression-free survival. SST receptor imaging using initially ^111^In-pentetreotide, and more recently, ^68^Ga-DOTATATE, which takes advance of the over-expression of SSTRs by NETs is the most sensitive method to image tumor location/extent in these patients. Lastly, numerous animal studies and non- prospective clinical studies, demonstrated that patients with advanced NETs expressing SSTRs could be treated with radiolabeled SST analogs with good antitumor effects. Recently, a large double -blind study in patients with advanced ileal carcinoid NETs confirmed this result leading to FDA approval for this approach using ^177^Lu labeled octreotate. The SST research shows that neuropeptide GPCRs can be used as oncotargets to detect and treat a human cancer.

A goal is to advance the cancer research on BB, NTS, and VIP. SST is inhibitory in nature and was initially used to inhibit secretions from NETs. Few effective therapies were available and octreotide effectively controlled the symptoms of patients with VIPomas, glucagonomas, GRFomas, insulinomas or gastrinomas. In contrast, BB, NTS, and VIP are stimulatory in nature and a substantial effort has been made to improve the half-life of peptide agonists in the blood and develop specific high affinity antagonists which are stable. SSTRs are present in over 90% of the NETs. BB, NTS, and VIPRs are not so universal in epithelial cancers. By precision medicine, the overexpression of GRPCRs for BB, NTS or VIP in the biopsy specimen will dictate which GPCR should be targeted. To illustrate if the tumor overexpresses NTSR1, the patient may be treated with SR48692 alone or in combination with another drug. Additional clinical trials are needed in BB, NT, and VIP research so that these peptide GPCR's can be used as oncotargets to treat epithelial cancers of the breast, colon, lung, and prostate.

## Author contributions

TM, IR-A and RJ are responsible for the writing of the manuscript and the figure preparation.

### Conflict of interest statement

The authors declare that the research was conducted in the absence of any commercial or financial relationships that could be construed as a potential conflict of interest.
